# Relationship Between Cerebrovascular Reactivity and Cognition Among People With Risk of Cognitive Decline

**DOI:** 10.3389/fphys.2021.645342

**Published:** 2021-05-31

**Authors:** Donghoon Kim, Timothy M. Hughes, Megan E. Lipford, Suzanne Craft, Laura D. Baker, Samuel N. Lockhart, Christopher T. Whitlow, Stephanie E. Okonmah-Obazee, Christina E. Hugenschmidt, Matthew Bobinski, Youngkyoo Jung

**Affiliations:** ^1^Department of Biomedical Engineering, University of California, Davis, Davis, CA, United States; ^2^Department of Radiology, University of California, Davis, Davis, CA, United States; ^3^Department of Internal Medicine, Wake Forest School of Medicine, Winston-Salem, NC, United States; ^4^Department of Radiology, Wake Forest School of Medicine, Winston-Salem, NC, United States

**Keywords:** cerebrovascular reactivity, cerebral blood flow, hypercapnia, cognition, arterial spin labeling

## Abstract

Vascular risk factors (e.g., obesity and hypertension) are associated with cerebral small vessel disease, Alzheimer’s disease (AD) pathology, and dementia. Reduced perfusion may reflect the impaired ability of blood vessels to regulate blood flow in reaction to varying circumstances such as hypercapnia (increased end-tidal partial pressures of CO_2_). It has been shown that cerebrovascular reactivity (CVR) measured with blood-oxygen-level-dependent (BOLD) MRI is correlated with cognitive performance and alterations of CVR may be an indicator of vascular disfunction leading to cognitive decline. However, the underlying mechanism of CVR alterations in BOLD signal may not be straight-forward because BOLD signal is affected by multiple physiological parameters, such as cerebral blood flow (CBF), cerebral blood volume, and oxygen metabolism. Arterial spin labeling (ASL) MRI quantitatively measures blood flow in the brain providing images of local CBF. Therefore, in this study, we measured CBF and its changes using a dynamic ASL technique during a hypercapnia challenge and tested if CBF or CVR was related to cognitive performance using the Mini-mental state examination (MMSE) score. Seventy-eight participants underwent cognitive testing and MRI including ASL during a hypercapnia challenge with a RespirAct computer-controlled gas blender, targeting 10 mmHg higher end-tidal CO_2_ level than the baseline while end-tidal O_2_ level was maintained. Pseudo-continuous ASL (PCASL) was collected during a 2-min baseline and a 2-min hypercapnic period. CVR was obtained by calculating a percent change of CBF per the end-tidal CO_2_ elevation in mmHg between the baseline and the hypercapnic challenge. Multivariate regression analyses demonstrated that baseline resting CBF has no significant relationship with MMSE, while lower CVR in the whole brain gray matter (*β* = 0.689, *p* = 0.005) and white matter (*β* = 0.578, *p* = 0.016) are related to lower MMSE score. In addition, region of interest (ROI) based analysis showed positive relationships between MMSE score and CVR in 26 out of 122 gray matter ROIs.

## Introduction

Neurodegeneration and cerebrovascular dysfunction in the brain coexist in most cases of dementia. They may also interact with one another and can cause cognitive decline (Silvestrini et al., 2006; Kapasi et al., 2017). Moreover, it has been shown that vascular cognitive impairment and Alzheimer’s disease (AD) share common risk factors such as hypertension and diabetes mellitus (Silvestrini et al., 2006; Montine et al., 2014). Cerebral blood flow (CBF) is one of the brain perfusion parameters representing the blood supply to the brain, and measurements of CBF response to a stimulus may provide a useful index of cerebral vascular function. In the case of dynamic functional change, CBF change can be evoked for various causes such as brain activations and vasoactive challenges; it returns to its steady state baseline by cerebral auto-regulation to maintain proper brain perfusion.

Baseline CBF alterations have been observed in the early stages of AD. Decreased CBF correlates with the severity of cognitive symptoms showing the potential contribution of CBF alteration to cognitive decline (Bracko et al., 2020). Therefore, brain vascular health is undoubtedly associated with cognitive performance among people with AD. Cerebrovascular reactivity (CVR) may explain the connection between cognitive function and brain vascular health since CVR is an indicator of the compensatory dilatory capacity of blood flow in the brain in response to vasoactive stimuli or vasoactive challenges. The hemodynamic change of CVR in the brain corresponds to its physiological condition such as normal condition or hypercapnia challenge. Thus, CVR may be a more sensitive brain perfusion parameter compared to baseline CBF.

During the hypercapnic challenge, the brain’s CO_2_ level is elevated by CO_2_ inhalation. The elevated CO_2_ level dilates cerebral blood vessels and correspondingly leads to vasodilation in the brain. This is because the increased CO_2_ level of the interstitial compartment and in the endothelial cells decreases interstitial pH level, resulting in relaxation of vascular smooth muscle cells (Liu et al., 2019). Based on this molecular mechanism, endothelial dysfunction can lead to impaired CVR and, consequently, cause CBF alteration. In addition, from various existing literatures, vascular endothelial dysfunction has shown to be closely related to cognitive decline (Gates et al., 2009; Dickstein et al., 2010; Leszek et al., 2012; Gazewood et al., 2013; Cahill-Smith and Li, 2014). Therefore, investigating CVR during hypercapnia challenge and its relationship with cognitive function may enable us to identify an early imaging biomarker by revealing the relationship between brain vascular perfusion and cognitive functions.

There are several methods measuring CBF using MRI. MRI has advantages in spatial specificity and non-ionizing radiation exposure. Among MRI technologies, arterial spin labeling (ASL) and blood-oxygen-level-dependent (BOLD) are two representative methods measuring CBF because they are non-invasive and easily repeatable. Compared to ASL, the BOLD signal has a better signal to noise ratio and more accessibility on conventional MRI scanners than ASL. However, BOLD is not a direct measurement of CBF. BOLD signal is a complex combination of CBF, cerebral blood volume, and cerebral metabolic rate of oxygen (CMRO2). Therefore, measuring both CVR and CBF from BOLD signal is inevitably affected by the variations of each parameter, which is not neglectable (Catchlove et al., 2018b). Specifically, when there are differences in baseline CBF or CMRO2, BOLD signal gives inaccurate metabolic information, which causes more ambiguity (Bangen et al., 2009). Furthermore, a previous study reported that BOLD signal is more sensitive to vascular tension than perfusion (Halani et al., 2015). In contrast, ASL quantitatively measures CBF directly.

A hypercapnic challenge can be induced by breath-holding, rebreathing exhaled gas, or inhalation of CO_2_-enriched air. Breath-holding prevents the participants from eliminating CO_2_, inducing the hypercapnic challenge by increasing partial pressure of CO_2_ (pCO_2_). Rebreathing exhaled gas using a rebreathing reservoir also increases pCO_2_, which results in the hypercapnic challenge. Both methods do not require an external source of CO_2_. This characteristic is quite advantageous because delivering a designated gas mixture to a participant inside the MRI scanner using an external source of CO_2_ requires several conditions. To be specific, all components of the gas delivery system inside of the MRI room must be MR-compatible. In addition, the breathing equipment has to be small enough to fit inside of a head coil with minimal discomfort. It also should be able to adjust the gas mixture without causing subject motion. However, breath-holding and rebreathing have their own drawbacks that make both methods unsuitable to achieve standardized hypercapnia for CVR measurements. Aside from breath-holding time, other factors such as individual metabolic rate and the size of lungs also affect pCO_2_ level (Fierstra et al., 2013). Thus, suitable breath-holding time is highly variable across participants (Markus and Harrison, 1992; Totaro et al., 1999; Fierstra et al., 2013). For the rebreathing method, oxygen is continuously consumed from the reservoir, and therefore it changes CBF continuously. However, the oxygen level should be kept consistent to measure CVR (Fierstra et al., 2013). Therefore, inhalation of CO_2_-enriched air using a proper MR-compatible gas delivery system is the most adequate method.

The purpose of this study was to investigate if CVR is related to cognitive performance among older adults who have an increased risk of cognitive decline. We hypothesize that hypercapnic CVR is closely related to cognitive performance for older adults even though they do not have any severe neurodegenerative or neurological diseases other than mild cognitive impairment (MCI). We also hypothesize that CVR is a more sensitive measurement than baseline CBF regarding cognitive performance among them. To investigate these, we conducted a region of interest (ROI) based pseudo-continuous ASL (PCASL) study using a programmable computer-controlled gas delivery system (RespirAct, Thornhill Medical, North York, Canada) that induced a hypercapnia challenge. In addition, we employed a Mini-mental state examination (MMSE) test to examine the relationship between cognitive performance and both baseline CBF and hypercapnic CVR.

## Materials and Methods

### Study Participants

Seventy-eight subjects (56 women/22 men) were recruited and enrolled in the Wake Forest Alzheimer’s Disease Research Center (ADRC) Clinical Core cohort for this Institutional Review Board (IRB) approved study. The subjects include 24 diagnosed with MCI (diagnosed according to NIA/Alzheimer’s Association criteria) and 54 with normal cognition. The MCI subjects included 10 amnestic single-domain MCI (aMCI), eight amnestic multi-domain MCI (mdaMCI), two non-amnestic multi-domain MCI (mdnaMCI), and four non-amnestic single domain MCI (sdnaMCI). Global cognition was assessed with the MMSE test (MMSE score: 28.41 ± 1.43; range: 25–30). The mean age was 69 years (*SD* = 7.18). The mean years of education was 15.6 years (*SD* = 2.45). The participants include 38 adults with hypertension and 12 with Type 2 diabetes. The mean body mass index (BMI) was 27.29 (*SD* = 5.12). Tables 1 and 2 show the characteristics and risk factor stratification of the study population, respectively.

**Table 1:** Characteristics of the study population.

Characteristics	All	MCI	Normal cognition	Statistical test between MCI and normal cognition
Sex: Male/Female, *n*	22/56	9/15	13/41	*p* = 0.3454
Hypertension, *n*	38	15	23	*p* = 0.1682
Type 2 Diabetes, *n*	12	4	8	*p* = 0.8343
Race: White/Black or AA, *n*	67/11	20/4	47/7	*p* = 0.9352
BMI	27.29 (5.12)	26.76 (5.37)	27.71 (5.14)	*p* = 0.4684
MMSE score	28.41 (1.43)	27.71 (1.55)	28.74 (1.23)	*p* = 0.0064
Age, *years*	69 (7.18)	71.5 (7.25)	67.72 (6.96)	*p* = 0.0352
Years of education, *years*	15.6 (2.45)	14.13 (2.03)	16.20 (2.40)	*p* = 0.0002
Hypercapnic CVR in gray matter, *%/mmHg*	2.22 (0.71)	2.09 (0.76)	2.27 (0.68)	*p* = 0.3110
Hypercapnic CVR in white matter, *%/mmHg*	1.94 (0.75)	1.76 (0.74)	2.03 (0.75)	*p* = 0.1380
Baseline CBF in gray matter, *mmHg*	35.87 (9.66)	33.42 (6.88)	36.97 (10.54)	*p* = 0.0816
Baseline CBF in white matter, *mmHg*	19.75 (6.03)	18.73 (4.28)	20.20 (6.64)	*p* = 0.2460

Values are means (s.d.) or numbers. Chi-squared test and *t*-test were used for categorical variables and quantitative variables, respectively, for statistical tests between mild cognitive impairment (MCI) and normal cognition groups.

**Table 2:** Risk factor stratification of the study population by cognition status.

	MCI	Normal Cognition
None	8	27
Hypertension only	12	19
Type 2 Diabetes only	1	4
Both	3	4

Values are numbers.

Exclusion criteria included significant neurologic diseases that might affect cognition, such as AD, stroke, Parkinson’s disease, multiple sclerosis, or recent severe head injury with loss of consciousness for more than 30 min within the last year, or with permanent neurologic sequelae. Participants with organ failure, active cancer, clinical depression, psychiatric illness, current use of strongly anticholinergic or sedative medications, use of anticonvulsant for a seizure disorder, current use of insulin, history of substance abuse, or heavy alcohol consumption within previous 10 years, use of pacemakers, aneurysm clips, artificial heart valves, ear implants, metal/foreign objects in the eyes, and inability to lie on the scanner bed for 40 min, or claustrophobia were also excluded.

### MRI Acquisition and Hypercapnic Challenge

Participants underwent MRI including T1-weighted and PCASL sequences. PCASL images were acquired during the normal condition and hypercapnic challenge. Experiments were performed on a 3 T Siemens Skyra (MRI), using a 32-channel head coil. T1-weighted anatomical images were obtained using a three-dimensional volumetric magnetization prepared rapid gradient echo (MPRAGE) sequence with resolution of 1 mm × 1 mm × 1 mm (TR = 2300 ms; TE = 2.98 ms; TI = 900 ms; flip angle = 9°; FOV = 240 × 256). Baseline CBF maps were obtained with multi-phase PCASL (Jung et al., 2010; 2D EPI; TR = 4,000 ms; TE = 11 ms; matrix = 70 × 56; flip angle = 90°; FOV = 21 cm × 16.8 cm; number of slices = 36; slice thickness = 4 mm, labeling duration = 1.8 s; post-labeling delay = 1.2 s; eight PCASL phases). A dynamic PCASL was obtained during a respiratory challenge (2D EPI; TR = 4,000 ms; TE = 25 ms; matrix = 64 × 64; flip angle = 90°; FOV = 21 cm × 21 cm; number of slices = 24; slice thickness = 5 mm, labeling duration = 1.8 s; post-labeling delay = 1.2 s).

**Figure 1:**
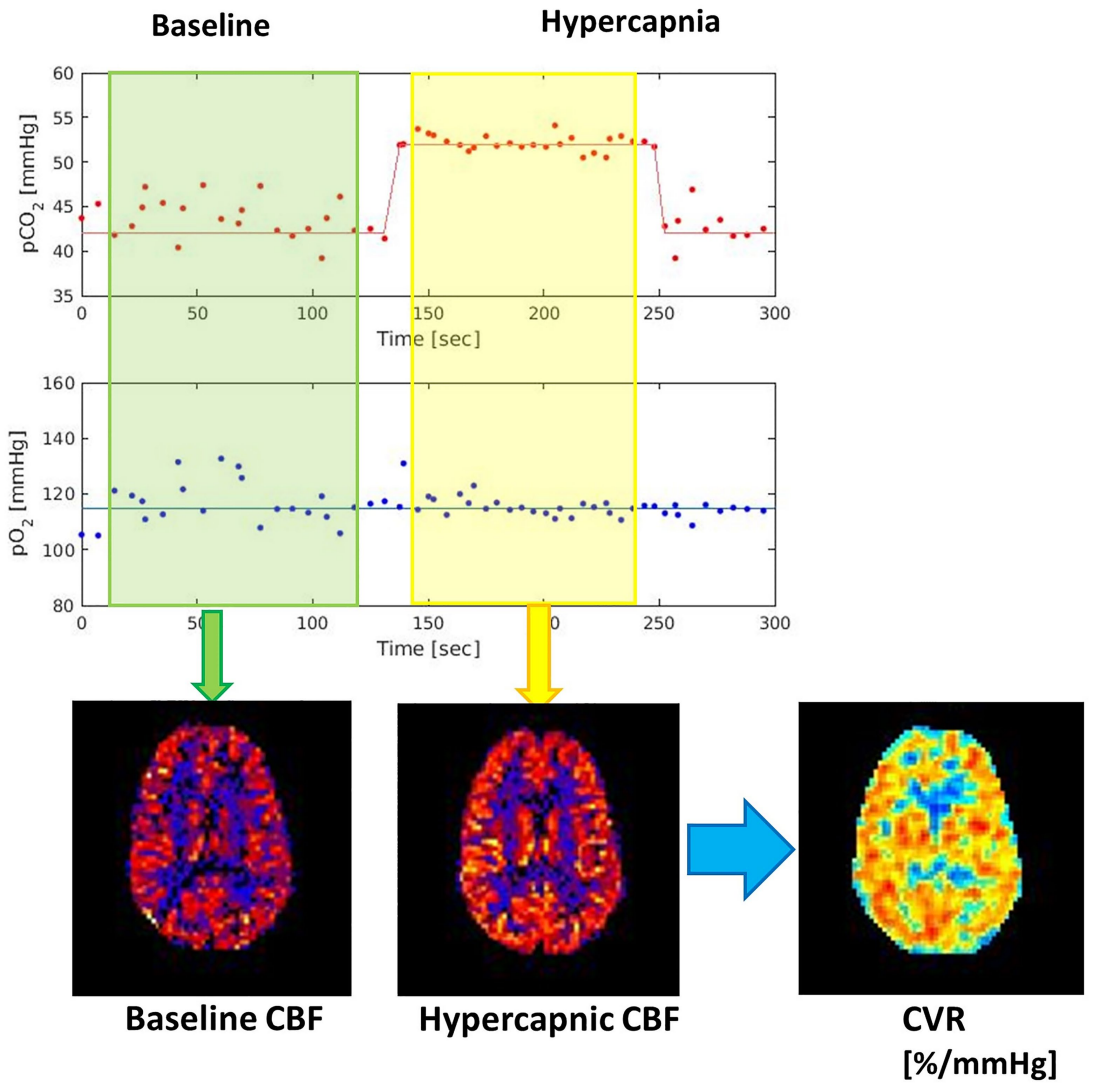
Vascular reactivity measurement using a hypercapnia paradigm. Per-voxel based cerebrovascular reactivity (CVR) images are generated by calculating % changes of blood flow per mmHg of partial pressure of CO_2_ (PCO_2_).

The effective post-labeling delays from the end of PCASL labeling to the acquisition of 2D slices range from 1.2- to 2-s depending on the slice locations. Inferior slices are acquired first and have shorter delays. These relatively short delay times are shown effective to reflect CVR impairment (Zhuang et al., 2021). The first volume of PCASL scans was acquired after playing two dummy TRs without any RF or gradient spoiling. The effective TR of the first volume was 2 * TR + labeling direction + the effective post-labeling delay, ranging from 11 to 11.8 s depending on the slice locations. The long TR produces proton density images which represent the net magnetization. The dynamic PCASL acquisition time was 5 min, including 2 min of baseline scan, 2 min of the hypercapnic period, and 1 min recovery back to baseline. The hypercapnia challenge was achieved by inhalation of CO_2_-enriched air using an MRI-compatible computer-controlled gas control system. Hypercapnia was employed by applying a target end-tidal PCO_2_ (P_ET_CO_2_) 10 mmHg above resting values while maintaining end-tidal PO2. The mean resting P_ET_CO_2_ level of entire participants was 39.70 mmHg (*SD* = 3.68; range: 30.1–48.1 mmHg). During the hypercapnic periods, the mean P_ET_CO_2_ level of entire participants was 49.52 mmHg (*SD* = 3.88; range: 41.3–58.2 mmHg). A dynamic PCASL scan was acquired to monitor CBF changes during resting and hypercapnic periods, and baseline and hypercapnic CBF maps were generated after averaging volumes from each period (Figure 1).

### Image Processing and CVR

Structural T1 image processing included a file conversion to Neuroimaging Informatics Technology Initiative (NIFTI) format, normalization, and segmentation. Brain normalization and segmentation were performed using SPM12 and CAT12 tools (Penny et al., 2011; Gaser and Dahnke, 2016). PCASL image processing included NIFTI conversion, head motion correction, partial volume correction, normalization, and segmentation. For head motion correction, we employed the *spm_realign* function from SPM12 (Penny et al., 2011). ASL images were quantified using a kinetic model (Buxton et al., 1998) and converted to absolute units of CBF (ml/100 g/min). The CSF signal estimated from the first volume of PCASL scans was used as a reference value (Wong, 2005) during the quantification. To correct partial volume effects that are attributed to the signal cross-contamination, we employed a kernel-based algorithm (Asllani et al., 2008). Partial volume corrected PCASL images were registered onto T1-weighted images and then normalized onto the reference image: Montreal Neurological Institute (MNI) template.

Cerebrovascular reactivity was calculated as the voxel-wise difference between baseline resting CBF and CBF during a hypercapnic challenge and divided by the change of P_ET_CO_2_ in mmHg using Equation 1.


(1) $$CVR=100\times \frac{CBF_{Hypercapnia}-CBF_{rest}/CBF_{rest}}{\Delta P_{ET}CO_{2}}$$

Regional CBF and CVR were obtained using the Automated Anatomical Labeling (AAL) atlas and the Johns Hopkins University (JHU) atlas for the gray matter and white matter, respectively. In addition, the mean CBF and CVR values of each ROI were computed. Outlier detection was not applied based on CVR or CBF values.

### Statistical Analyses

Multivariate linear regression analyses were employed to investigate the relationships between CBF or CVR in both whole brain gray and white matter with MMSE score, adjusted for covariates: age, sex, race, BMI, years of education, hypertension status, diabetic status, hypertension medication status, diabetic medication status, and either gray matter or white matter volume.

Similarly, in each ROI of the gray and white matters, the relationship between regional CBF or CVR and MMSE score was investigated with the same covariates. Covariate-adjusted residuals were then obtained from the overall model fit. All analyses were conducted in Matlab R2020a (Mathworks, Natick, MA, United States) using a value of *p* = 0.05 to designate significance. The false discovery rate method with a tuning parameter, Lambda of 0.5, was used to test multiple hypotheses across ROIs (122 ROIs for gray matter and 48 ROIs for white matter).

To examine if the relationship between MMSE score and CBF or CVR is driven by cognitive status, a sensitivity analysis that evaluated potential interactions with cognitive status was also performed with covariates of age, sex, race, BMI, years of education, hypertension status, diabetic status, hypertension medication status, and diabetic medication status.

## Results

### CBF in the Gray Matter and White Matter

Without covariate adjustment, CBF in the gray matter showed a negative association with age (*p* = −0.484, *p* = 0.001). With adjustment for other covariates but not gray matter volume, CBF in the gray matter was also negatively associated with age (*β* = −0.472, *p* = 0.006). Additional adjustment for the total gray matter volume attenuated this relationship (*β* = −0.370, *p* = 0.042). The regression results showed that CBF in the gray matter and white matter was not associated with MMSE score with (*p* = 0.787 in gray matter and *p* = 0.781 in white matter) or without (*p* = 0.362 in gray matter and *p* = 0.919 in white matter) adjustments for covariates.

### CVR in the Gray Matter and White Matter

**Figure 2:**
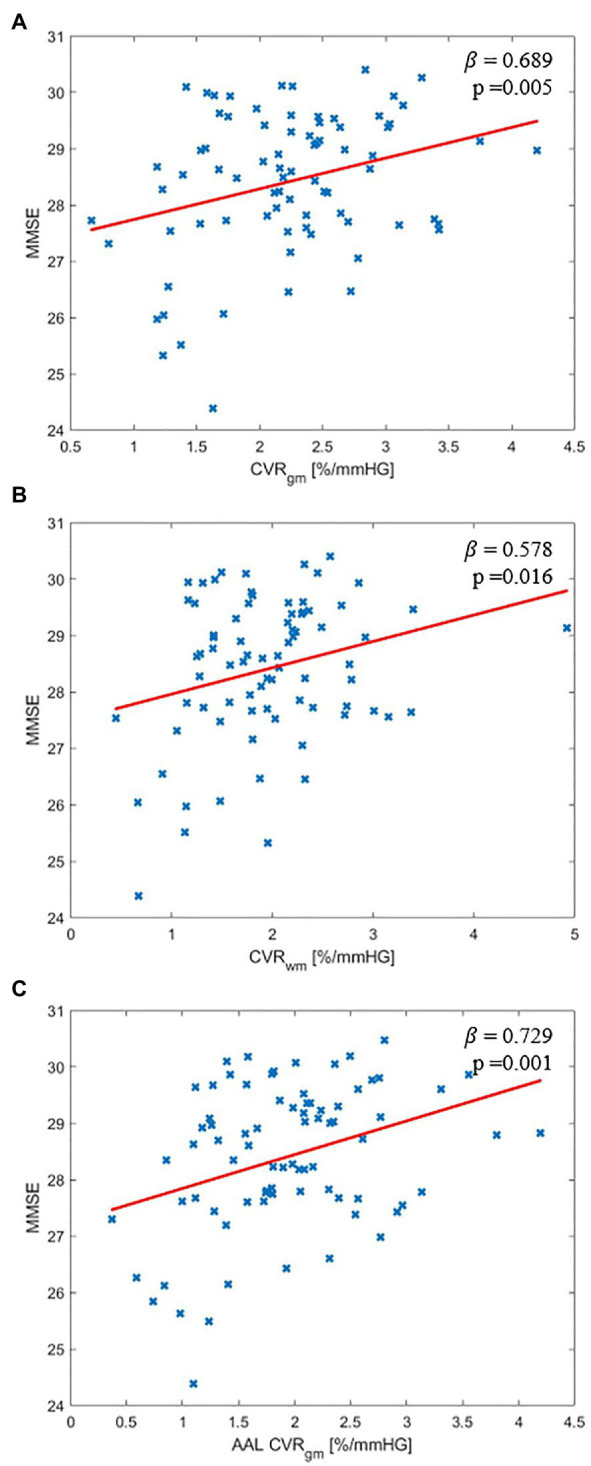
Covariate adjusted relationship between Mini-mental state examination (MMSE) score and CVR: **(A)** Whole brain gray matter, **(B)** Whole brain white matter, and **(C)** Volume weighted average of reported 26 gray matter region of interests (ROIs).

**Figure 3:**
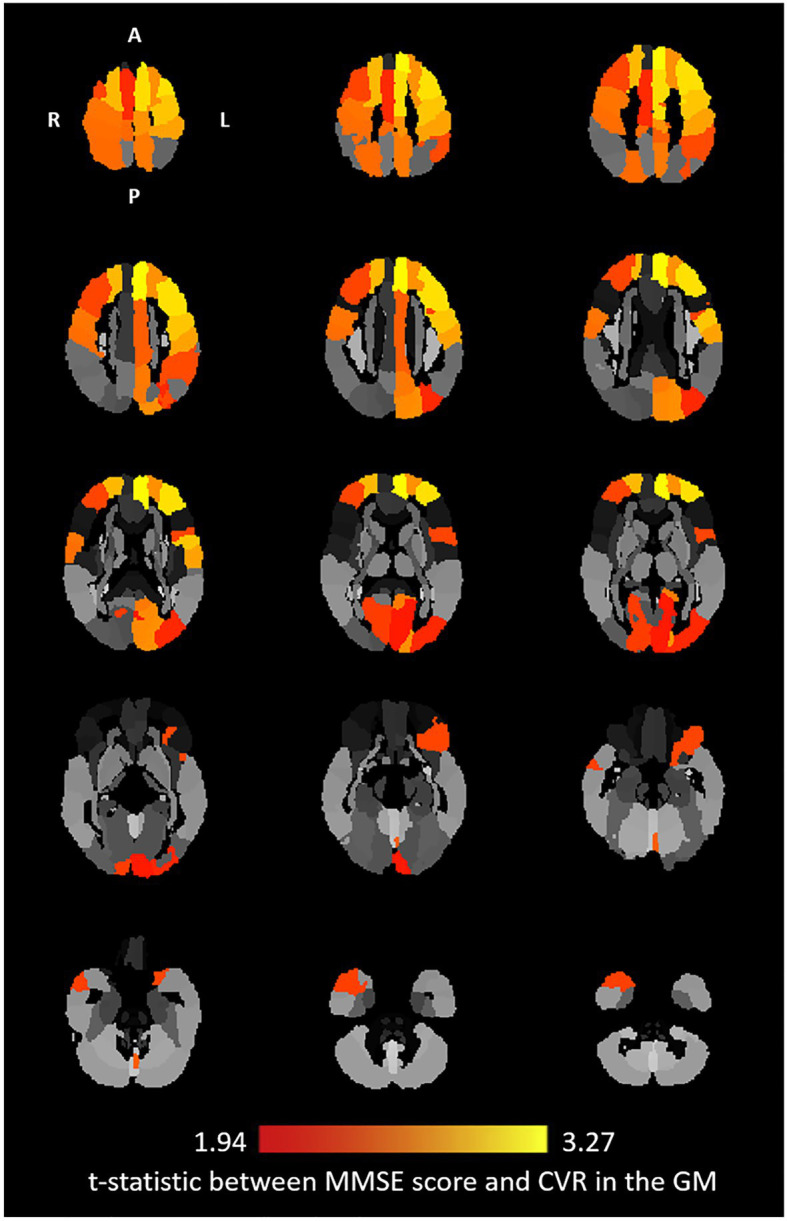
Covariates adjusted t-statistic: axial view of the relationship between CVR and MMSE score at the regional level in the gray matter. Twenty-six ROIs were reported from the multivariate linear regression where corrected value of *p* < 0.05 [Automated Anatomical Labeling (AAL) ROIs: Left Precentral, Right Precentral, Left Superior Frontal, Right Superior Frontal, Left Middle Frontal, Right Middle Frontal, Left Inferior Frontal Operculum, Left Inferior Frontal Orbital, Left Superior Motor, Right Superior Motor, Left Superior Medial Frontal, Left Middle Cingulum, Left Calcarine, Right Calcarine, Left Cuneus, Left Superior Occipital, Left Middle Occipital, Left Postcentral, Right Postcentral, Right Superior Parietal, Left Inferior Parietal, Left Precuneus, Left Central Paracentral Lobule, Right Central Paracentral Lobule, Right Middle Temporal Pole, and Left Vermis 6]. The brightest yellow color corresponds to a maximum t-statistic among the regions.

Unadjusted linear regression analysis between MMSE score and CVR in the gray matter showed a positive relationship (*p* = 0.534, *p* = 0.023). However, there was no statistically significant relationship between MMSE score and CVR in the white matter (*p* = 0.427, *p* = 0.064). Multivariate linear regression analysis of CVR in the whole brain gray matter or white matter revealed positive relationships between MMSE score and CVR (gray matter: *β* = 0.689, *p* = 0.005, white matter: *β* = 0.578, *p* = 0.016) with the adjustments for age, sex, BMI, years of education, hypertension status, diabetic status, hypertension medication status, diabetic medication status, and total gray matter or white matter volume (Figures 2A,B). Multivariate linear regression analysis of regional CVR in gray matter demonstrated that there were statistically significant relationships between higher MMSE score and higher CVR in 26 regions out of 122 gray matter ROIs (Figure 3). Figure 2C illustrates the relationship between MMSE score and CVR in the gray matter ROIs from the covariate-adjusted linear fit model. The CVR value of each ROI was weighted by its volume data. Note that in Figure 2, MMSE scores were adjusted with covariate-adjusted residuals. From ROI analysis in the white matter, 10 out of 48 ROIs showed a positive relationship between MMSE score and regional CVR, but none of these relationships reached statistical significance after adjustment for multiple comparisons.

### MMSE Score and Covariates

Linear regression between MMSE score and years of education showed a positive correlation (*β* = 0.232, *p* = 0.0003). This positive correlation also appeared in all multivariate linear regression analyses in this study with *p* value of 0.05 to designate significance. There were no other covariates significantly associated with MMSE score.

### Cognition Subgroups

Mild cognitive impairment and normal cognition groups showed different MMSE scores (*t*-test value of *p* = 0.006). Additional multivariate linear regression including the cognition status as a covariate showed that the relationship between the whole brain CVR and MMSE was nearly identical in both gray matter (*p* = 0.009) and white matter (*p* = 0.034). In addition, there was no significant interaction effect between cognition status and CVR regarding MMSE score.

## Discussion

Our results showed that CVR in both gray matter and white matter of the whole brain was positively associated with cognitive performance among the subjects with an age range of 56–89 years without any significant neurologic diseases that affect cognitive decline other than MCI. In contrast, there was not any statistical relationship between baseline CBF and cognitive performance. The positive correlation between CVR and cognitive performance was also reported in previous studies with a variety of diseases, such as AD (Silvestrini et al., 2006; Cantin et al., 2011; Richiardi et al., 2015), dementia (Silvestrini et al., 2006; Lee et al., 2007), multiple sclerosis (Metzger et al., 2018), Moyamoya disease (Calviere et al., 2010), endothelial dysfunction (Lavi et al., 2006), and atherothrombotic disease (Haratz et al., 2015). Therefore, CVR may have an important role in the early stage of these neurological diseases that may impair auto-regulation of CBF and lead to cognitive decline. Our result regarding the baseline CBF on cognitive performance is inconsistent with some of the past findings. Our findings including such inconsistency revealed a number of important considerations about CBF and CVR with cognitive performance among people with risk of cognitive decline. First, some previous studies showed that reduced resting CBF was associated with cognitive decline (Meyer et al., 1988; Kogure et al., 2000; Spilt et al., 2005; Poels et al., 2008; Okonkwo et al., 2014; Sabayan et al., 2014; Iturria-Medina et al., 2016; Bracko et al., 2020). Second, many studies indicated that CVR decreases with advancing age (Reich and Rusinek, 1989; Tsuda and Hartmann, 1989; Lu et al., 2011; Flück et al., 2014). Third, the intrinsic nature of the link between CVR alteration and cognitive decline is still equivocal.

Previous studies reported a reduction in baseline CBF associated with advanced age and decreased cognitive function (Meyer et al., 1988; Kogure et al., 2000; Spilt et al., 2005; Poels et al., 2008; Okonkwo et al., 2014; Sabayan et al., 2014; Iturria-Medina et al., 2016; Bracko et al., 2020). However, most studies regarding the reduced CBF on the cognitive decline were reported with an incidence of disease, such as AD (Kogure et al., 2000; Okonkwo et al., 2014; Iturria-Medina et al., 2016; Bracko et al., 2020), dementia (Meyer et al., 1988; Spilt et al., 2005), and endothelial dysfunction (Sabayan et al., 2014). One previous study in healthy subjects reported that lower CBF was associated with poorer cognitive performance including information processing and executive function, but this association disappeared after brain volume correction (Poels et al., 2008). Also, different APOE genotypes can impact the relationship between CBF and cognition differently (Wierenga et al., 2012, 2013). Adding to this literature, cognitive function may be more related to the ability of CBF regulation rather than its baseline alteration based on our results.

Although many studies reported that CVR in the brain decreases with advancing age (Reich and Rusinek, 1989; Tsuda and Hartmann, 1989; Lu et al., 2011; Flück et al., 2014), age-related alteration in CVR is still vague. This is attributed to the fact that there are conflicting results, as other studies found no age-related differences in CVR (Davis et al., 1983; Ito et al., 2002; Schwertfeger et al., 2006; Murrell et al., 2013). Due to these inconsistencies, some studies suggested that CVR reduction with age may be underestimated (McKetton et al., 2018). In this study, there was no significant relationship between age and CVR in the whole brain gray and white matters. Based on our results, CVR response in the whole brain may not be significantly altered by age in the range of 56–89 years. However, further investigation is needed because CVR reduction with advancing age may occur in a smaller proportion of voxels in the brain.

The intrinsic nature of the association between CVR alteration and cognitive function still needs to be investigated even though we demonstrated that lower CVR is correlated with lower cognitive performance. There are several mechanisms that can potentially explain this association, including vascular stiffening and endothelium related underproduction of vasodilators. This is because advancing age leads to vascular stiffness, which can cause decreased reactivity of cerebral blood vessels (Desjardins, 2015). In addition, aging alters the L-arginine-NO pathway and consequently reduces endothelium-dependent vasodilation and this leads to decreased CVR (Taddei et al., 2006; Catchlove et al., 2018a). Another potential mechanism is reduced BOLD activation resulted from reduced CVR (Williams et al., 2021). This is because reduced BOLD activation was associated with cognitive decline in various neurological diseases, such as multiple sclerosis and cerebral small vessel disease (Mainero et al., 2004; Rocca et al., 2014; Atwi et al., 2018; Williams et al., 2021). Vascular steal may also be related to cognitive function with reduced CVR. A redistribution of CBF to adjacent active areas due to a progressive arterial narrowing causes reduced or negative CVR (Conklin et al., 2010; Williams et al., 2021). Vascular steal phenomenon was associated with cognitive decline among healthy adults (Conklin et al., 2010). Furthermore, in patients with Moyamoya disease, this phenomenon is commonly observed with cognitive decline (Conklin et al., 2010; Williams et al., 2021).

Several limitations of this study should be recognized. The inclusion of cognitive status did not appreciably change the results suggesting that the observed relationships were similar for cognitively normal and MCI participants. This observation was supported by a lack of interactive effects between cognitive status and MMSE on CVR in the regression models. Further investigation is needed with a larger cohort to investigate the contribution of cognition status to CVR response. APOE genotype data were not included in the regression models. Different APOE genotypes can impact the relationship between CBF baseline and cognition differently (Wierenga et al., 2012, 2013). Also, MCI subtype was not included in the statistical models in this study due to the small sample size of each subtype. In addition, only a MMSE score was used to represent the cognitive performance of each participant. MMSE score may not be ideal to represent individual’s overall cognitive function because MMSE is more specialized for language and memory (Bak and Mioshi, 2007; Mamikonyan et al., 2009). Lastly, using pre-defined brain ROIs might have lower statistical sensitivity compared to voxel-wise analysis if an affected region is smaller than an ROI. Our current study cohort may not be large enough for a voxel-wise analysis, but our future study will include whole-brain voxel-wise statistical analyses on a larger cohort to identify specific voxel clusters showing a statistically significant relationship between CVR and cognitive performance.

In conclusion, this study demonstrated that lower CVR in the whole brain white matter and gray matter and in regional gray matter were related to lower MMSE score. In addition, MMSE has a positive correlation with years of education, while MMSE score does not have any significant relationships with other covariates, such as age, sex, BMI, the status of diabetes or hypertension, and gray matter or white matter volume. On the other hand, the MMSE score is not statistically associated with the baseline CBF. Therefore, impaired CVR in the reported gray matter regions in this study and the whole brain white matter may be early imaging biomarkers revealing the relationship between cerebrovascular perfusion and cognitive functions. Based on the mechanisms regarding vascular stiffness and endothelium related underproduction of vasodilators, vaso-protective therapies may have an important role in preventing cognitive decline among older adults with a risk of cognitive decline.

## Data Availability Statement

The raw data supporting the conclusions of this article will be made available by the authors, without undue reservation.

## Ethics Statement

The studies involving human participants were reviewed and approved by WFU Health Sciences IRB Office, 336-716-4542. The patients/participants provided their written informed consent to participate in this study.

## Author Contributions

DK performed the experiments and wrote the manuscript supervised by YJ. TH, SC, CH, and MB contributed to the interpretation of the results. ML and CW contributed to image data processing. SC and LB recruited the participants. SL performed image data quality control and management. SO-O performed the analysis of cognitive tests. YJ planned the experiments and interpreted the results. All authors contributed to the article and approved the submitted version.

### Conflict of Interest

The authors declare that the research was conducted in the absence of any commercial or financial relationships that could be construed as a potential conflict of interest.
