# Corrigendum: Relationship between cerebrovascular reactivity and cognition among people with risk of cognitive decline

**DOI:** 10.3389/fphys.2022.1020999

**Published:** 2022-09-23

**Authors:** Donghoon Kim, Timothy M. Hughes, Megan E. Lipford, Suzanne Craft, Laura D. Baker, Samuel N. Lockhart, Christopher T. Whitlow, Stephanie E. Okonmah-Obazee, Christina E. Hugenschmidt, Matthew Bobinski, Youngkyoo Jung

**Affiliations:** ^1^ Department of Biomedical Engineering, University of California, Davis, Davis, CA, United States; ^2^ Department of Radiology, University of California, Davis, Davis, CA, United States; ^3^ Department of Internal Medicine, Wake Forest School of Medicine, Winston-Salem, NC, United States; ^4^ Department of Radiology, Wake Forest School of Medicine, Winston-Salem, NC, United States

**Keywords:** cerebrovascular reactivity, cerebral blood flow, hypercapnia, cognition, arterial spin labeling

In the published article, there was an error. We inaccurately wrote Equation 1.

A correction has been made to **Materials and Methods**, “*Image Processing and CVR*,” Equation 1. The corrected sentence appears below:

“

CVR=100×CBFHypercapnia−CBFrestCBFrest∆PETCO2 (1)

”

The authors apologize for this error and state that this does not change the scientific conclusions of the article in any way. The original article has been updated.

